# Seqotron: a user-friendly sequence editor for Mac OS X

**DOI:** 10.1186/s13104-016-1927-4

**Published:** 2016-02-17

**Authors:** Mathieu Fourment, Edward C. Holmes

**Affiliations:** The ithree institute, University of Technology Sydney, Sydney, Australia; Marie Bashir Institute for Infectious Diseases and Biosecurity, Charles Perkins Centre, School of Biological Sciences and Sydney Medical School, The University of Sydney, Sydney, Australia

**Keywords:** Sequence editor, Alignment, Phylogenetics

## Abstract

**Background:**

Accurate multiple sequence alignment is central to bioinformatics and molecular evolutionary analyses. Although sophisticated sequence alignment programs are available, manual adjustments are often required to improve alignment quality. Unfortunately, few programs offer a simple and intuitive way to edit sequence alignments.

**Results:**

We present Seqotron, a sequence editor that reads and writes files in a wide variety of sequence formats. Sequences can be easily aligned and manually edited using the mouse and keyboard. The program also allows the user to estimate both phylogenetic trees and distance matrices.

**Conclusions:**

Seqotron will benefit researchers who need to manipulate and align complex sequence data. Seqotron is a Mac OS X compatible open source project and is available from Github https://github.com/4ment/seqotron/.

## Background

State-of-the-art methods of multiple sequence alignment such as MUSCLE [1] and MAFFT [2] are usually used to automatically generate alignments. Unfortunately, these methods can be inaccurate when the input sequences are highly dissimilar or when sequencing errors have been incorporated. Hence, it is important to visually inspect any sequence alignment prior to subsequent analysis to detect and correct potential errors. There are a large number of sequence editors that allow sequence alignments to be displayed, including Se-Al [3], Jalview [4], SeaView [5], Mesquite [6] and UGENE [7]. However, only a few (e.g. Se-Al) provide a simple and intuitive way to edit sequence alignments. In addition, it is often problematic to convert files into different file formats, even though a wide variety of formats are required for different applications.

Herein, we present a user-friendly application for visualizing, aligning, and manually editing genomic and protein sequences, and for converting between a variety of file formats. Alignments can be generated automatically using the MUSCLE [1] and MAFFT [2] packages and the quality of the alignment can be visually inspected and manually corrected using simple mouse-based and keyboard-based operations. In addition, Seqotron allows the computation of distance matrices and the inference of phylogenetic trees through the Physher program [8].

## Implementation

Seqotron is written in Objective-C and uses Cocoa, the native application programming interface for the Mac OS X operating system.

## Results and discussion

**Fig. 1:**
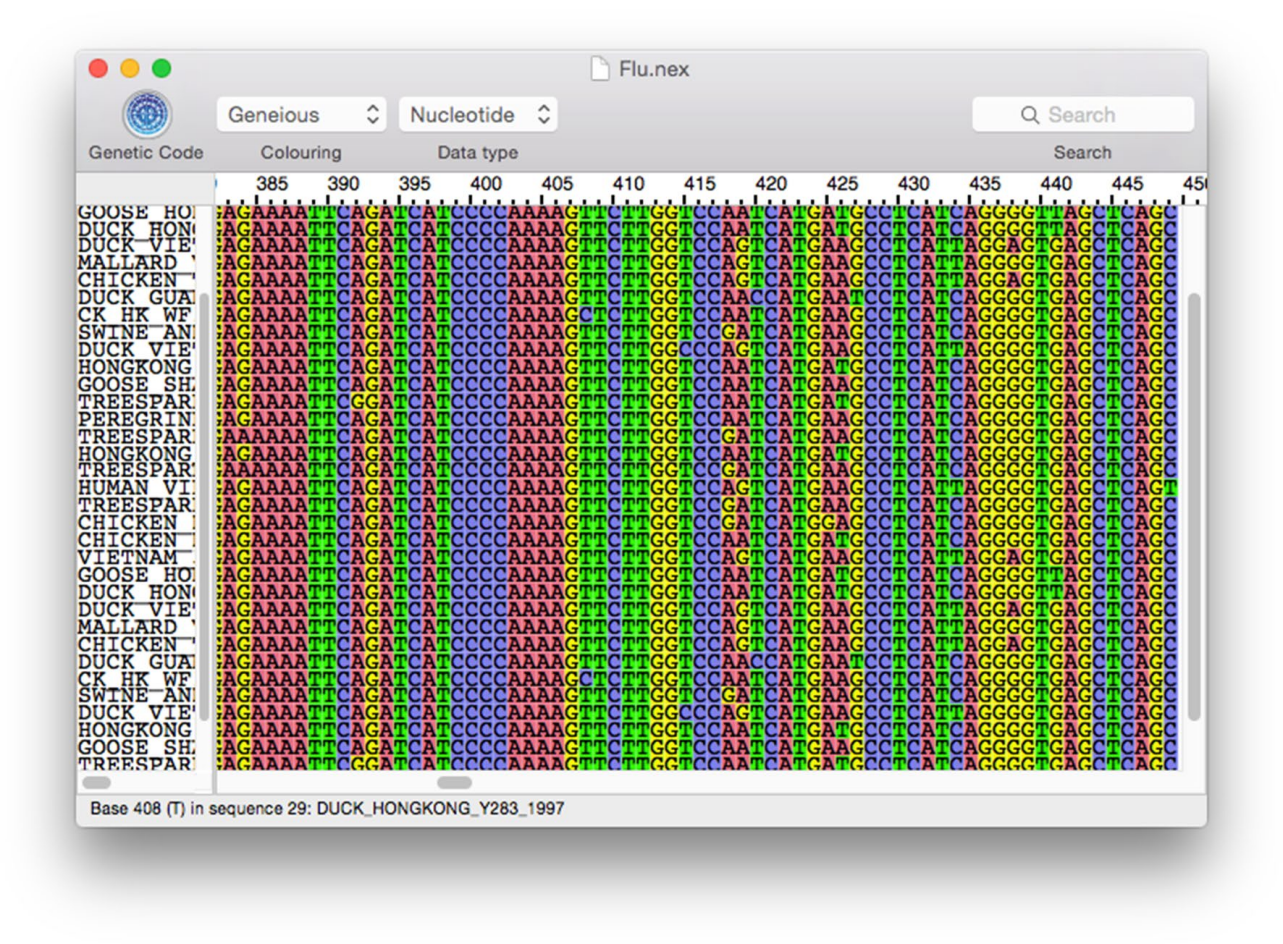
Visualisation of a nucleotide alignment in Seqotron. This screenshot displays a region of an alignment

Seqotron is designed for visualizing, aligning, and editing nucleotide and amino acid sequences (Fig. 1). Unaligned sequences and multiple sequence alignments can be imported and exported in a wide range of formats including: FASTA, NEXUS, NEWICK, PHYLIP, MEGA, Clustal, NBRF, Stockholm, and GDE. The sequence viewer can display sequences using different preset color schemes, such as the standard ClustalX coloring scheme. In addition, Seqotron allows the user to create personalized coloring schemes using a color editor. Sequences can be aligned or realigned using MUSCLE [1] and MAFFT [2]. The alignment of protein-coding DNA sequences can also be achieved using their amino acid translation during the alignment process before reverting to DNA sequences [9]. One or a group of sequences can be manually edited by dragging regions of the alignment using the mouse in a similar way to Se-Al. In addition, selected regions can be removed in an intuitive way using the keyboard. A nucleotide sequence alignment can easily be temporarily translated according to any genetic code available, while allowing the user to simply revert to the original nucleotide sequences. Manual editing of translated sequences is also available. Another function that is useful for the analysis of segmented genomes (such as found in some viruses including influenza) in a phylogenetic context is the ability to concatenate sequences with identical names. This option is provided when several files are open at the same time.

**Fig. 2:**
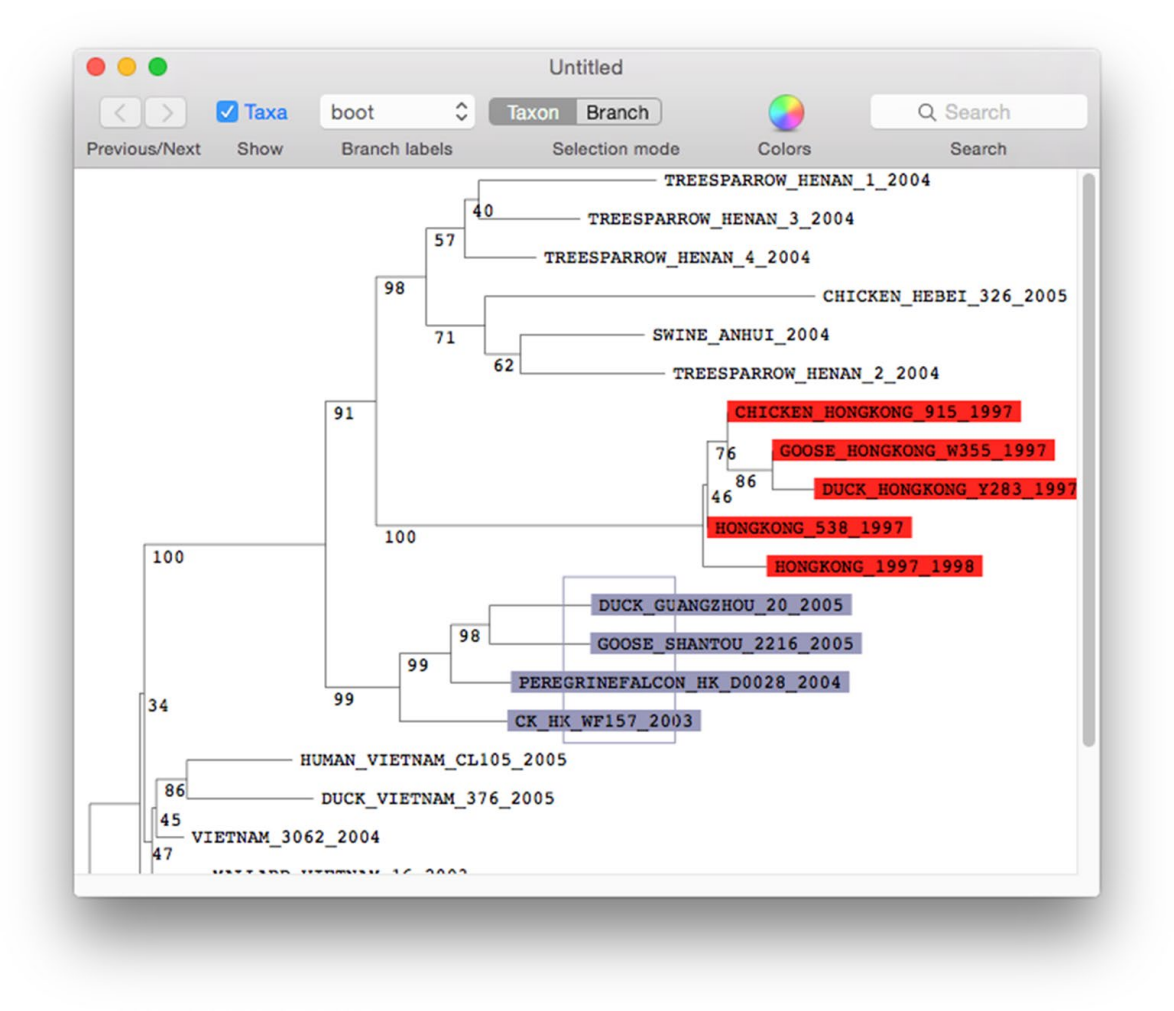
Visualisation of a phylogenetic tree in Seqotron. This screenshot displays a neighbour joining phylogenetic tree inferred from the data set in Fig. 1. Bootstrap values computed from 100 replicates are shown next to each branch. The tree was built using Physher, a program included in Seqotron

Given an accurate alignment of homologous sequences, it is natural to investigate the evolutionary history of the underlying organisms using phylogenetic methods. Seqotron allows the inference of phylogenetic trees using Physher [8] from both amino acid and nucleotide sequences using distance-based (neighbor-joining and UPGMA) and maximum likelihood methods. Statistical support for each branch can be assessed through non-parametric bootstrapping and jackknifing. These resampling methods can be parallelized across multiple cores for higher efficiency. Physher’s binaries are packaged with the Seqotron application and therefore does not require installing any third-party programs or libraries. Seqotron provides a tree viewer (Fig. 2) to display newly generated trees or trees stored on file in NEXUS or NEWICK formats. The tree viewer provides additional functionalities such as taxa coloring, search by taxon name, re-rooting, node rotation, printing, and exporting to NEWICK-based text and PDF files. Another common task is to extract a subset of sequences for further investigation based on a their evolutionary relationship. To this end, Seqotron allows the selection of sequences through the tree viewer. In the case of segmented genomes a single tree can be used to select the same sequences in different alignments.

Finally, Seqotron supports natively the Quick Look technology that enables the Finder to display a quick preview of an alignment file and other useful information such as the number of sequences and the alignment length.

A comparison of the features available in Seqotron and other editors is provided in Table 1. Seqotron uses the native language of Mac OS X and therefore tends to be more memory efficient than editors written in other programming languages. Indeed, a common problem with programs written in Java is that they are prone to consume a large amount of memory. In some cases, when the amount of memory required to run the program exceeds a certain threshold, the user has to adjust the maximum heap size in a trial and error fashion and restart the application. We have compared the memory consumption of Seqotron to other programs using an alignment in a FASTA file containing 2813 sequences and 2277 sites on an iMac running Mac OSX 10.11 with a 3.2 GHz Intel Core i5 processor and 16 gigabytes of memory. The physical memory determined with the program top is reported. Se-al was not included since it does not run on Intel-based Apple computers. Seqotron is slightly more memory efficient than SeaView, requiring 54 and 85 megabytes (MB), respectively. Mesquite and Jalview showed the largest memory footprint requiring 2.98 gigabytes (333 MB when the data set is loaded from a NEXUS file) and 446 MB of memory, respectively. We also profiled the memory consumption and the speed of each program using the Instruments tool during the inference of a neighbor-joining tree. The same alignment was used to infer the tree and the total runtime also includes the calculation of an un-corrected pairwise distance matrix. Seqotron estimated the phylogenetic tree in 37 s and the memory peaked at 115 MB. Sea View was significantly slower: 5 min 56 s and the memory peak is higher with 769 MB during the inference of the tree. After the alignment was read as a NEXUS file, Mesquite calculated the tree in 23 min and its memory peak was 439 MB. Jalview used 635 MB and required more than 6 h to complete the analysis.

**Table 1:** Comparison of Seqotron to other sequence editors

	Seqotron	Jalview	SeaView	Se-Al	Mesquite
Concatenate sequences	Yes	–	Yes	Yes	Yes
Mouse-based alignment	Yes	Yes	–	Yes	–
Transalign^a^	Yes	–	–	Yes	–
Temporary translation	Yes	–	–	Yes	–
Alignment zooming	Yes	–	–	Yes	–
Distance matrix	Yes	–	–	–	Yes
Loading tree formats	NEXUS, NEWICK	NEWICK	–	–	NEXUS
Estimating trees	NJ^b^, UPGMA^c^, ML^d^	NJ, UPGMA	NJ, MP^e^, ML	–	NJ, MP
Tree resampling	Bootstrap, jackknife	–	Bootstrap	–	–

^a^Alignment of protein-coding DNA sequences using their amino acid translation

^b^Neighbor-joining (NJ)

^c^Unweighted pair-group method using arithmetic averages (UPGMA)

^d^Maximum likelihood (ML)

^e^Maximum parsimony (MP)

## Conclusions

We have presented an open source, memory efficient, and user-friendly desktop application to automatically or manually align and edit multiple nucleotide and amino acid sequences. Seqotron also provides the option to estimate phylogenetic trees and distance matrices. We aim to add more functionalities in the future, such as creating a plugin mechanism and algorithms for searching sequence motifs.

### Availability and requirements

Project name: Seqotron.

Project home page: https://github.com/4ment/seqotron/.

Operating system: Macintosh OS X (Intel) version 10.8 and higher.

Programming language: Objective-C/Cocoa.

License: GNU GPL version 3.

Any restrictions to use by non-academics: None.

## Abbreviations

MUSCLE: multiple sequence comparison by log-expectation
MAFFT: multiple alignment using fast Fourier transform
PHYLIP: phylogeny inference package
MEGA: molecular evolutionary genetics analysis
NBRF: National Biomedical Research Foundation
GDE: genetic data environment
UPGMA: unweighted pair group method with arithmetic mean
